# Impact of Car Traffic on Metal Accumulation in Soils and Plants Growing Close to a Motorway (Eastern Slovakia)

**DOI:** 10.3390/toxics10040183

**Published:** 2022-04-07

**Authors:** Margita Kuklová, Ján Kukla, Helena Hniličková, František Hnilička, Ivica Pivková

**Affiliations:** 1 Institute of Forest Ecology, Slovak Academy of Sciences, 960 01 Zvolen, Slovakia; kukla@ife.sk (J.K.); ivica.pivkova@ife.sk (I.P.); 2 Department of Botany and Plant Physiology, Czech University of Life Sciences Prague, 165 00 Prague, Czech Republic; hnilickova@af.czu.cz (H.H.); hnilicka@af.czu.cz (F.H.)

**Keywords:** heavy metals, soil contamination factors, soil-plant transfer coefficients, traffic-related pollution

## Abstract

The paper evaluates the impact of car transport on the distribution and accumulation of Zn, Cu, Pb and Cd in soils, as well as in the vegetation near a newly built R4 motorway Košice-Milhosť (Slovakia). Samples were taken from surface humus layer (litter) and 0–5, 10–20 and 20–30 cm mineral layers of Cambisol and Luvisol, as well as from assimilatory organs of *Fraxinus excelsior*, *Quercus cerris*, *Quercus rubra*, *Negundo aceroides* and *Anthriscus sylvestris* growing in the segments of geobiocoenosis *Querci-Fageta Typica*. The concentrations of total Zn and Cu were determined using SensAA AAS and the total concentrations of Cd and Pb using an instrument iCE 3000 Series AAS-F. Contamination factor (CF) values showed that surface humus layer of both soil units is moderately contaminated with Zn (1 ≤ CF ˂ 3), low contaminated with Cu (CF ˂ 1) and considerably contaminated with Pb and Cd (3 ≤ CF ˂ 6). Contamination of the surface humus layer of Luvisol with Pb is very high (CF > 6), while in the case of mineral layers with Zn and Cu it is low (CF ˂ 1). The mineral layers of Luvisol are moderately contaminated with Pb and Cd (1 ≤ CF ˂ 3) and Cambisol layers with Zn, Cu, Pb and Cd. For the group of 5 tested plants, higher values of toxic elements in the leaves were observed on Luvisol compared to Cambisol. However, only Cu conconcentrations in Luvisol significantly correlated with Cu concentrations in plants (r > 0.4 or r < 0.6). The same can be said for Zn concentrations in Cambisol (r > 0.8). The best indicator of the environment polluted by car traffic appears to be *A. sylvestris*. Transfer coefficients (TC ˃ 1) revealed that this species concentrated the most Zn and Cu on Luvisol and close to 1 are also the TC values found for Cu in *F. excelsior* and *Q. cerris* leaves taken on Luvisol. Lead is accumulated most efficiently in *N. aceroides* leaves and Cd in *A. sylvestris* leaves regardless of soil unit. Compared to background values, the total concentrations of trace elements in soils and plants were significantly higher and point to the pollution of forest ecosystems already in the initial stage of motorway operation.

## 1. Introduction

Soils and plants can receive large amounts of risk elements from different anthropogenic sources [1,2], but especially from car traffic emissions [3,4,5]. Therefore, accumulation of heavy metals in the plant-soil system should be systematically and thoroughly investigated, especially near major roads [6]. Various studies have shown severe contamination of soil and vegetation with toxic elements near roads, but also a sharp decrease in concentrations with distance from roads [7,8,9,10]. Lead and cadmium levels in soils and vegetation mostly decrease with increasing distance from the roadside [11]. Studies also found that Pb burden in the environment is strongly related to the vehicular traffic density. Overall, surface layer of soil is characterized by the highest heavy metal concentration [12]. Additionally, studies have shown that high metal concentrations alter litter decomposition due to impaired biological functions [13,14].

Also, atmospheric deposition of heavy metals is widely documented and has been connected to adverse ecological and health impacts. Feng et al. [15] studied the influence of atmospheric deposition on the soil–rice system in a typical urban agglomeration region in Hunan Province of southern China. Their results showed that Cd and Pb concentrations in rice grains were mainly from soil, but Cd and Pb from the atmospheric deposition should be a focus of attention. Similarly, Ouyang et al. [16] carried out the research on uptake of atmospherically deposited Cd by leaves of vegetables using subcellular localization by NanoSIMS. They found out that Cd concentrations in vegetable leaves treated with suspensions of CdS nanoparticles reached up to 11.0 and 39.8 mg kg^−1^, which were higher than the critical leaf concentration for toxicity.

Heavy metals such as Cu, Zn, Pb and Cd contained in contaminated soils have a high potential bioavailability to soil biota, plants and humans [17,18]. Presence of Cd near roads is being attributed to dust from the combustion of petrol. The concentrations of Ag, Ba, Cd, Pb, Sb, V, and Zn in nano particles were strongly associated with diesel fuel, while Cu, Mn, and Sr in particles < 0.1 μm were more strongly associated with gasoline [19]. One of the potential sources of Zn can be wear and corrosion of vehicle parts (brakes, tyres, radiators, bodies and engine parts) [20]. Tyres typically contain 1–2% ZnO, and more than 4000 t Zn year^−1^ is released via tyre debris in the European Union [21]. Atmospheric emissions of Pb were at their peak during the Industrial Revolution, and the leaded gasoline period in the second half of the twentieth century [22].

In Slovakia until the 1970s, one liter of petrol contained approximately 0.6 g of lead, and motor vehicles released approximately 120,000 tons of toxic lead into the atmosphere. Unleaded petrol has been used since 1985 and has resulted in a reduction in the amount of lead in car exhaust to around 20,000 tons per year. This lead comes from unleaded petrol, which still contains up to 0.005 g Pb l^− 1^ [23]. Additional contamination comes from corrosion of Pb wheel weights. Its residence time in the environment is very long [9]. 

Copper mobility along the soil profile, bioavailability for root uptake and consequently phytotoxicity threshold for crops depend on ecological characteristics of soils [24,25]. Also, zinc accumulation is influenced both by soil properties and plant species. Most often, Zn is absorbed by plants in proportion to its concentration in the soil [26]. A portion of the heavy metals can be converted into plant-accessible forms by various processes in the soil. Study from Hunan Province of China points out the importance of accumulated elemental ratios that can determine the source of heavy metal contamination in soil, which is important for the prevention and control of heavy metal soil pollution and remediation at plot scale [27]. Of all the potentially toxic elements in soils, either endogenous or exogenous, Cd is one of the most dangerous trace elements because of its high solubility and toxicity [28]. Plants can accumulate significant amounts of Cd in their aboveground organs [29,30], which is related to high mobility of Cd in the natural environment [31]. This element, which is non-essential for plants, accumulates in the soil and subsequently becomes toxic to all living organisms [32]. 

In addition, the leaves and exposed parts of plants generally act also as absorbers of polluted air. Therefore, knowledge of heavy metal transfer to plants and screening of potential plant accumulators is a basic prerequisite for successful environmental remediation [33]. The ability of plants to handle excessive amounts of heavy metals is variable [34]. The oak tree health survey, for example, showed increased defoliation and insect damage near the motorway, and the beech tree health survey found poorer crown condition close to the motorway [5]. Beeches growing the closest to the source of air pollution (acid deposition) in turn had a higher incidence of bark damage [35]. Some plant species can deposit risk elements in cell walls or vacuoles, reducing their toxicity. For example, *Betula pendula* can successfully accumulate Cu (271 mg kg^−1^) and Zn (283 to 1649 mg kg^−1^) and *Silene vulgaris* (159 mg kg^−1^) Zn in their aboveground organs [36]. The metal accumulation is significantly affected also by plant morphology. Hence, in recent years, many studies have focused on the concentration and distribution of heavy metals in soil-plant system [7,8]. In addition to Pb and Cd, believed to be an inseparable part of dust issued by motor vehicles, Zn and Cu are also among the most common soil and vegetation pollutants in the area of communication routes [34,37,38]. 

The motorway can affect plants over long distances [7,9]. Environmental pollution of Kosice Basin (eastern Slovakia) is caused mainly by metallurgical, chemical and other processing industries, production of thermal and electric energy as well as car traffic, especially along a newly built motorway R4 Košice-Milhosť. Emissions mainly include particulate matter SOx NOx, CO, F and heavy metals. The highest levels of Pb were found in dust of the Košice urban agglomeration [39]. The assessment of the level of air pollution through the lichen (*Xanthoria parietina*) showed that the amount of toxic elements in this city depends on the distance from the main source of pollution (Zn ˃ Cu ˃ Cd), the US Steel factory [40].

We assume that forest ecosystems located along the newly built R4 expressway may already contain heavy metals in the initial phase of operation. For this reason, the aim of this work is to verify the hypothesis of possible contamination of soil and plants with trace elements based on the evaluation of: (1) the amount of Zn, Cu, Pb and Cd in soils and assimilatory organs of selected plant species, (2) the level of soil contamination by contamination factors, (3) the level of transfer of the risk element from soil to plants using transfer coefficients, (4) the ability of the tested plant species to accumulate risk elements.

The data obtained can be useful in the long term in assessing the development of accumulation and transfer of heavy metals from soil and air into specific plant species, as well as in screening potential plant accumulators and proposing remedial measures.

## 2. Materials and Methods

### 2.1. Study Area

**Figure 1:**
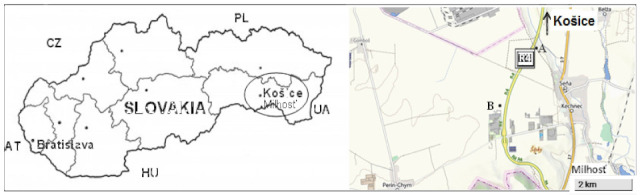
Location of study areas A and B near the R4 motorway Košice-Milhost’.

The research was carried out near the motorway R4 Košice-Milhos’ (south-eastern Slovakia) during the summer aspect of phytocoenoses, in the first week of August 2016. The construction of the motorway lasted from 2012 to November 2013, and the years 2014–2016 already characterized the full operation of the motorway. The motorway passes through an agricultural landscape in which only a few smaller, anthropically strongly influenced segments of forest geobiocenoses have been preserved. We selected the two best-preserved forest segments (study areas A and B), that are located 30 m from the motorway and are about 2.5 km apart. Within each study area, 3 sampling sites were selected, each with an area of 100 m^2^ (Figure 1).

The classification of geobiocoenoses was made according to Zlatník [41], while soils were classified according to the WRB IUSS [42], (Table 1).

Segment of forest ecosystem A represents a sparse pasture forest formed by the co-dominant species *Quercus robur* L. and *Quercus cerris* L. with sporadic occurrence of *Quercus rubra* L., *Fraxinus excelsior* L., *Populus nigra* L. and *Robinia pseudoacacia* L. The woody plant extending into the main crown level of the stand is *Negundo aceroides* Moench. The herbaceous layer with a total coverage of 90–100% consists of 71 species, of which 12 grasses. Mesotrophic grasses predominate, especially *Poa angustifolia* L., *Bromus arvensis* L. and *Festuca ovina.* From other species are abundant *Achillea milefolium* L., *Anthriscus sylvestris* (L.) Hoffm., *Galium mollugo* L., *Geranium robertianum* L., *Picris hieracioides* subsp. *hieracioides* L., *Taraxacum officinale* agg., *Urtica dioica* L. subsp. *dioica, Veronica chamaedrys* subsp. *chamaedrys* L. and *Torilis arvensis* (Huds.) Link.

The vertically highly differentiated segment of forest ecosystem B borders on an artificially constructed canal draining stagnant rainwater from cultivated agricultural land. The main crown layer of the stand consists of *Fraxinus excelsior* L. *Quercus rubra* L. and *Populus x canadensis* Moench, rare is *Robinia pseudoacacia* L. The woody plants extending into the main level of the stand are *Quercus cerris* L. and *Negundo aceroide*s Moench and in lower layer of the stand there are *Fraxinus excelsior* L., *Quercus cerris* L. An unevenly distributed herbaceous layer reaching a cover of 30–60% consists of 21 species. Mesotrophic species dominate, especially *Moehringia trinervia* (L.) Clairv., *Anthriscus sylvestris* (L.) Hoffm., *Geum urbanum* L. and *Viola reichenbachiana* Jord. ex Boreau. The arrangement of the experiment can be seen in Table 2.

### 2.2. Soil Characteristics

Table 3 lists general characteristics of soils of the studied areas. Katoskeletal Cambisol of forest ecosystem A is a dusty clay soil with a skeletal concentration increasing significantly downwards. The upper 5 cm layer is neutral, rich in humus, with a favourable ratio of C:N. Katostagnic Luvisol of forest ecosystem B is moderately acid, predominantly silty loam and skeleton-free. The upper 5 cm layer is humus-rich with a C:N ratio between 11 and 12. The signs of weak redox processes begin at a depth of 37 cm.

### 2.3. Soil Analyses

Surface humus samples were taken in triplicate, each from randomly selected mini-plots with an area of 0.1 m^2^ and also mineral soil samples were taken in triplicate from layers 0–5 cm, 10–20 cm and 20–30 cm after excavation of the pits. The samples were air-dried and passed through a sieve with a mesh size of 2 × 2 mm. The particle-size distribution of the fine earth fraction dispersed by means of sodium hexametaphosphate and ultrasound was determined by laser particle sizer Analysette 22 (Fritsch, Germany). The values of soil reaction were determined potentiometrically by a digital pH meter Inolab pH 720 (WTW, Weilheim, Germany) and the total concentration of C and N using NCS analyser FLASH 1112 (Hanau, Germany).

Subsamples of soils intended for the determination of quasi total metals were ground down (<0.001 mm) using a Fritsch planetary micro-mill (Idar-Oberstein, Germany). The ground soil samples were mineralized with concentrated HNO_3_ mixed with 2 mL of deionised water at 190 °C for 15 min in speedwave MWS-2 microwave pressure digestion system (Berghoff, Germany). The concentrations of total Zn and Cu were determined using SensAA AAS (GBC Scientific Equipment, Braeside, Australia) and the total concentrations of Cd and Pb using an instrument iCE 3000 Series AAS-F (Thermo Scientific, Cambridge, UK) according to STN EN ISO 11885: 2009 (75 7466).

### 2.4. Plant Analyses

The concentration of risk elements was determined in leaves of two native woody species (*F. excelsior* L., *Quercus cerris* L.) two exotic woody species (*Q. rubra* L., *N. aceroides* Moench) and one of the most common herbal apophytes *A. sylvestris* (L.) Hoffm., which occur in both forest ecosystems. Random sampling of plant leaves was carried out on 3 plots, each with an area of 100 m^2^. From each plant species, 50 leaves were taken in triplicate (Table 2).

Plant samples were washed carefully to remove any dust particles, oven-dried at 80 °C for 48 h and homogenized with a Fritsch planetary micro mill PULVERISETTE 7 classic line (˂0.001 mm). The total Zn, Cu, Pb and Cd concentrations were determined after mineralisation of plant samples by concentrated HNO_3_ mixed with 2 mL of deionised water at 190 °C for 15 min in a speedwave MWS-2 microwave pressure digestion system (Berghoff, Germany). The total Zn and Cu concentrations were determined using an instrument SensAA AAS (GBC Scientific Equipment, Braeside, Australia) and the total concentration of Cd and Pb using an instrument iCE 3000 Series AAS-F (Thermo Scientific, Cambridge, UK) according to STN EN ISO 11885: 2009 (75 7466).

### 2.5. Data Analysis

Data were interpreted using Statistica, Version 9.0, StatSoft (Tulsa, OK, USA). All values were expressed as arithmetic mean ± standard deviation (SD). The variability of risk elements in soils and leaves sampled near the motorway was assessed based on the results of ANOVA (*p* ≤ 0.05). The influence of each significant factor (soil-unit horizons, plant species) was then tested according to Fisher’s LSD test. The Pearson’s correlation coefficients were used to check the relationships between element concentrations in the soils and plants.

The soil pollution was evaluated by means of contamination factor (CF) expressed as a ratio of the average concentration of risk element in a soil sample to the median value in Slovak soils (Zn: 66.3 mg kg^−1^, Cu: 19 mg kg^−1^, Pb: 22 mg kg^−1^, Cd: 0.3 mg kg^−1^), reported by Čurlík [43]. The level of soil contamination was evaluated according to the following scale: CF ˂ 1 low contamination; 1 ≤ CF˂ 3 moderate contamination; 3 ≤ CF ˂ 6 considerable contamination; CF > 6 very high contamination, reported by Hakanson [44]. The degree of contamination (Cdeg) is defined as the sum of the CFs. It is used to assess polymetallic contamination for each sampling sites [45]. Four categories have been defined for Cdeg as follows: <8 = low level of contamination, 8–16 = moderate level of contamination, 16–32 = considerable level of contamination, and >32 = very high level of contamination [46].

The ability of plant species to absorb risk elements from soil and atmosphere is reflected in the values of transfer coefficients (TC) indirectly indicating the health status of the plants and the risk that these plants pose in the food chain of forest ecosystems. These TC express a ratio of the concentration of elements found in plant and soil [47]. The values ˃ 1 indicate that the plant species (accumulator) concentrates element, the values around 1 indicate that the plant species (indicator) shows the proportional relationships between metal concentrations in the soil, uptake and accumulation in plant parts, and the values ˂ 1 show that plant species (excluder) excludes the element from the uptake [48].

## 3. Results

### 3.1. Profile Distribution of Trace Elements in Soils

**Figure 2:**
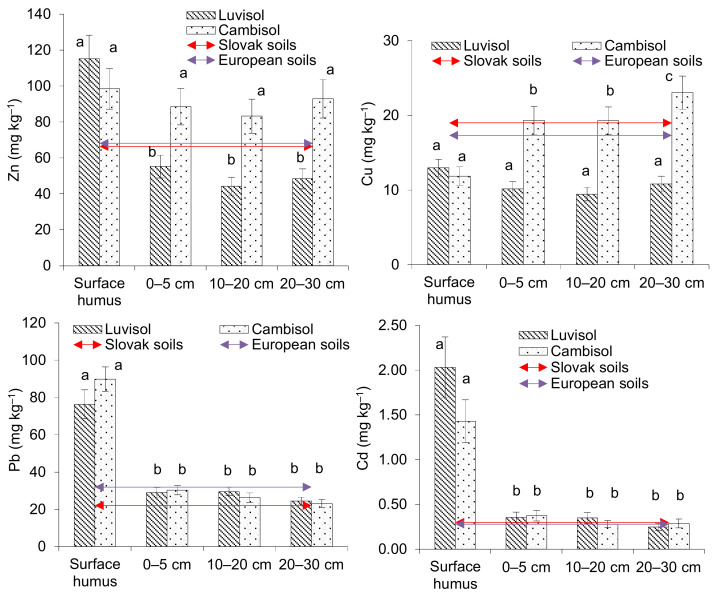
Concentrations of toxic elements in soils (*n* = 3). ANOVA, Fisher’s LSD test. Significantly different mean values (*p* ˂ 0.05) among soil-unit horizons are indicated by different letters (^a,b,c^). Median values of Slovak soils [43]; Mean values for the topsoil of Europe (Zn 68.1, Cu 17.3, Pb 32, Cd 0.28), after FOREGS 2005 [31].

The total concentrations of the risk elements found in soils of the studied forest ecosystems are shown in Figure 2. The concentration of zinc in the surface humus layer of Cambisol reaches 98 ± 11 mg kg^−^^1^ and is 1.1–1.2 times higher compared to the mineral layers. On the other hand, the concentration of Zn in the surface humus layer of Luvisol reaches 115 ± 13 mg kg^−^^1^ and is approximately 2.1–2.6 (2.3) times higher than in the mineral layers. The concentration of Zn in the mineral layers of Cambisol (on average 88 ± 10 mg kg^−^^1^) is 1.8 times higher compared to Luvisol, whose value reaches 49 ± 6 mg kg^−^^1^.

The concentration of copper in the surface humus layer of the studied Cambisol reaches 13 ± 1 mg kg^−^^1^, i.e., only 51–61 (57%) compared to the mineral layers. The surface humus layer of Luvisol contains 12 ± 1 mg Cu kg^−^^1^ and this value is about 1.2–1.4 (1.3) times higher than in the mineral soil layers. The concentration of Cu in the mineral layers of Cambisol (on average 21 ± 2 mg kg^−^^1^) is 2 times higher compared to Luvisol, whose value achieves 10 ± 1 mg kg^−^^1^.

Accumulation of lead in the surface humus layer of Cambisol reaches 90 ± 7 mg kg^−^^1^ and is 3.0–3.9 (3.4) times higher compared to the mineral layers. The surface humus layer of Luvisol contains 76 ± 8 mg Pb kg^−^^1^ and this value is 2.6–3.1 (2.8) times higher than in the mineral soil layers. The concentration of Pb in the mineral layer of Cambisol (on average 27 ± 2 mg kg^−^^1^) is approximately the same as for Luvisol mineral layer (on average 28 ± 2 mg kg^−^^1^).

Cadmium in the surface humus layer of Cambisol reaches 1.5 ± 0.2 mg kg^−^^1^ and is 3.8–5.1 (4.5) times higher compared to the mineral layers. The Luvisol surface humus layer contains 2.1 ± 0.3 mg Cd kg^−^^1^ and this value is 5.7–8.1 (6.4) times higher than in the mineral soil layers. The concentration of Cd in the Cambisol mineral layers (on average 0.3 ± 0.05 mg kg^−^^1^) is approximately the same as for Luvisol, whose value reaches 0.32 ± 0.05 mg kg^−^^1^.

Overall, the results show that surface humus layer contains significantly higher amounts of Zn, Pb and Cd (only Cambisol) compared to the mineral layers of the soils. In contrast, the concentration of Cu in the surface humus layer of Cambisol is significantly lower compared to the mineral layers and in the case of Luvisol this difference is not statistically significant (*p* > 0.05) (Figure 2).

### 3.2. Level of Soil Contamination

**Figure 3:**
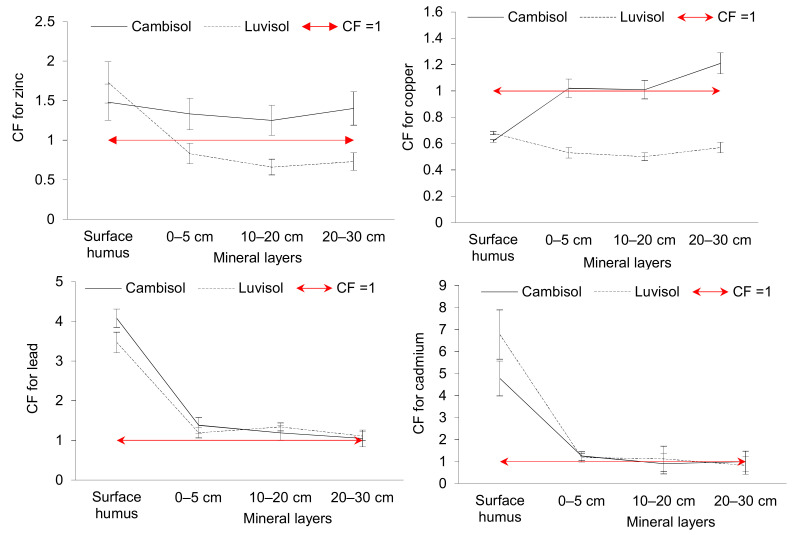
Soil contamination factors (arithmetic mean ± standard deviation) of trace elements (CF ˂ 1 = low contamination; 1 ≤ CF ˂ 3 = moderate contamination; 3 ≤ CF ˂ 6 = considerable contamination; CF > 6 = very high contamination).

Figure 3 shows the soil pollution rate evaluated using the contamination factor (CF) and calculated as a ratio of an average concentration of the risk element in the soil sample to the median value (Zn: 66.3 mg kg^−1^, Cu: 19 mg kg^−1^, Pb: 22 mg kg^−1^, Cd: 0.3 mg kg^−1^) found for Slovak soils [43].

CF values show that the surface humus layer of both soils is moderately contaminated with Zn (Cf 1.5–1.7), low contaminated with Cu (CF < 1) and considerably contaminated with Pb and Cd (Cf 3.5–4.8). On the other hand, contamination of Luvisol surface humus layer with Pb is very high (CF > 6). The mineral layers of Cambisol are moderately contaminated with Zn, Cu, Pb and Cd (Cf 1–1.5) and that of Luvisol with Pb and Cd (Cf 1.1–1.3). Contamination of Luvisol mineral layers with Zn and Cu is low (CF < 1).

The degree of polymetallic contamination (Cdeg) of soil samples on sampling sites showed that the surface humus samples had moderate level of contamination (sum of the CF of all elements is 11.0 on sites of Cambisol and 12.7 on sites of Luvisol). Mineral layers (0–30 cm) of both soils revealed low level of contamination (Cdeg ˂ 5).

### 3.3. Concentration of Trace Elements in Tested Plant Species

The range and mean concentrations of risk elements accumulated in the tested plant species are summarized in Table 4. The concentration of Zn, Cu and Cd in the leaves of species (except oaks) growing on Cambisol is usually slightly lower compared to the species growing on Luvisol. Higher concentration was found only for Zn in *Q. cerris* leaves and for Cu in *Q. rubra* leaves. On the other hand, a higher Pb concentration was in leaves of *F. excelsior*, *Q. rubra* and *N. aceroides* growing on Cambisol. Zinc and copper concentrations in *F. excelsior* leaves and Cu and Cd concentrations in *N. aceroides* leaves taken on both soils were also significantly different. On the other hand, the differences between average values of risk elements calculated for the groups of 5 tested plant species growing in different segments of forest ecosystems were not significant (ANOVA: Zn: F_(2,12)_ = 0.9922, *p* = 0.3992; Cu: F_(2,12)_ = 1.2401, *p* = 0.3239; Pb: F_(2,12)_ = 0.8780, *p* = 0.4407; Cd: F_(2,12)_ = 0.6911, *p* = 0.5199).

### 3.4. Transfer of Trace Elements in the Soil-Plant System

The values of transfer coefficients (TC) indicating the differences in the ability of plant species to accumulate risk elements in the studied environment are shown in Table 5. TCs found for the species growing on Luvisol (0.06–1.60) are generally higher compared to species on Cambisol (0.05–0.46). TCs higher than 1 were found only for *A. sylvestris* leaves on Luvisols (Zn 1.07, Cu 1.60) and close to 1 are also the TC values found for Cu in *F. excelsior* and *Q. cerris* leaves taken on Luvisol (reaching 0.90 and 0.94, respectively). In the case of Cambisol, the highest Zn and Cu accumulation was found in *Q. cerris* leaves (TC 0.46 and 0.44, respectively). Lead is accumulated most efficiently in *N. aceroides* leaves (TC 0.11 and 0.12) and Cd in *A. sylvestris* leaves regardless of soil unit. On the other hand, the lowest TC values were found for foliage of *F. excelsior* (Pb 0.06, Cd 0.10 on Luvisol and Zn 0.15 on Cambisol) and *N. aceroides* (Cd 0.09, Cu 0.22 on Cambisol and Zn 0.51 on Luvisol).

Correlations between Zn concentrations in *A. sylvestris*, *F. excelsior*, *Q. cerris*, *Q. rubra*, *N. aceroides* and Zn concentrations in Cambisol were significantly positive, while in case of species growing on Luvisol they were not significant (Table 6). The same can be said for Cu concentration of the plants growing on Luvisol. On the other hand, the correlations between Pb and Cd concentrations in plants and both soils were insignificant. This fact shows that Pb and Cd concentrations in plants are not controlled by their concentration in soils.

## 4. Discussion

### 4.1. Concentration of Trace Elements in Soils

Common range of Zn in soils is given in the range of 10–300 (on average 50) mg kg^−1^ [50]. Zinc is naturally present in all soils in typical background concentrations 10–100 mg kg^−1^ [51]. However, in the Netherlands, background concentrations of zinc in natural soils are in the wider range, from 6.4 to 150 mg kg^−1^ [52]. The total concentrations of Zn in the upper layers of Slovak soils range from 3 to 14,925 mg kg^−1^ with a characteristic interval of 52–86 mg kg^−1^ and a median value of 66.3 mg Zn kg^−1^ [43]. This value is only slightly higher than an average concentration of 64 mg Zn kg^−1^ for the unpolluted soils reported by Kabata-Pendias and Mukherjee [53]. The concentration of Zn found in the mineral layers of Cambisol constitutes only 83–93% and in the case of Luvisol only 44–55% of an average value stated by Kabata-Pendias and Mukherjee [53]. Considerably higher concentrations of Zn in soil samples than our values were found at a distance of 0.5 m from the Warsaw motorway (178.25–266.41 mg kg^−1^) [8]. 

In general, Cu accumulates more significantly in the surface horizon of soils due to its high affinity for organic matter, bioaccumulation and anthropogenic deposition of Cu. However, the concentration of Cu in the surface humus layer of Cambisol was slightly lower than in the mineral soil layers. Thus, the higher humus concentration in the topsoil was not reflected in a higher Cu bond. The mobility, bioavailability and toxicity of Cu are mainly controlled by sorption-desorption processes that occur in both organic and inorganic soil colloids. Therefore, not only the soil organic carbon, clay and oxides concentrations but also other properties (soil pH and the cationic exchangeable capacity) govern Cu availability in soil [54,55].

The total concentration of Cu determined in the upper layers of Slovak soils ranges from 1 to 22,360 mg kg^−1^ with a characteristic interval of 14–25 mg kg^−1^ and an average value of 19 mg Cu kg^−1^ [43]. Other authors state that an average Cu concentration in normal soils ranges from 2 to 100 mg kg^−1^ (on average 30 mg kg^−1^) [56,57,58]. Our values found in surface humus layer of the studied soils are higher than a lower limit of Cu range (11.4 and 17 mg kg^−1^) for unpolluted soils of Europe reported by Oorts [59]. Also, the concentration of Cu in the mineral layers of Cambisol is higher than the upper limit for unpolluted soils of Europe. The concentration of Cu in these layers is therefore only slightly affected by road traffic. On the other hand, the amount of Cu in the Luvisol mineral strata may indicate a Cu deficiency, as it represents only 70–80% of a lower limit of 14 mg kg^−1^ reported by Vaverková and Adamcová [30]. In general, concentration of Cu in the soil layers can be dependent on complex interactions between parent materials, physico-chemical properties of the soil but also possible exogenous inputs from agriculture or industry [60].

The common range of lead in soils is 2–200 mg kg^−1^, with a mean value of 10 mg kg^−1^ [50]. The mean value of total Pb for different soils is estimated as 27 mg kg^−1^ and in various soil groups varies within the range of 3–90 mg kg^−1^, being the highest in Cambisols and Histosols [31]. Lead concentration found in Slovak soils ranges from 3 to 2122 mg kg^−^^1^ with a characteristic interval of 17–31 mg kg^–1^ and a median value of 22 mg kg^−^^1^ [43].

Lead values found in the surface humus layer of the studied soils are about 2.8–3.3 times higher than the values reported by Kabata-Pendias [31], but up to 7–9 times higher compared to the Lindsay’s values [50]. It is therefore clear that Pb concentration in the studied soils is affected by car traffic. Average Pb concentration in the 0–30 cm mineral layer of both soils (26–27 mg kg^−^^1^) is almost identical to the value given by Kabata-Pendias [31], however about 1.2–1.3 times higher compared to median value for the upper layers of Slovak soils [43]. Lead values similar to ours were also found in the soil mineral layers near the Warsaw Expressway (E30) [8]. However, the total amount of Pb in upper layers of soils located near the Vilnius-Klaipėda motorway moved in the wider range of 7.88 to 54.27 mg kg^−1^ [61].

The common range of Cd in soils is 0.01–0.70 mg kg^−1^, with a mean value of 0.06 mg kg^−1^ [51]. Cadmium concentrations in upper horizons of Slovak soils vary in the range of 0.1 (detection limit) to 9.2 mg kg^−1^ with a characteristic interval of 0.2–0.4 mg kg^−1^ and a median value of 0.3 mg kg^−1^ [43]. The mean values of Cd found in the surface humus layer of the studied soils (Figure 1) are up to 24–34 times higher compared to the average value of 0.06 mg kg^−1^ reported by Lindsay [50], but only about 3.5–4.9 times higher compared to the average value 0.41 mg kg^−^^1^ stated by Kabata-Pendias [31]. The surface humus layer of both studied soils is therefore considerably contaminated. That is consistent with the finding that the highest Cd concentrations (2.26 mg kg^−1^) have the samples taken from topsoil layers 0.00–0.10 m [62]. On the other hand, Cd concentrations detected in the studied mineral soil layers are only negligibly higher in comparison with the median value of 0.3 mg kg^−1^ determined by Čurlík and Šefčík for Slovak soils [43]. Our values are only a little lower in comparison to Cd concentrations in the soils located in the immediate vicinity of the Warsaw Expressway (E30) (1.43 to 2.07 mg kg^−1^) [8]. On the other hand, our values were significantly lower than Cd content determined in the soils on the S51 road in Poland (0.015 mg kg^−1^) [63]. The total amount of Cd in the upper soil layers (0.096 to 1.19 mg kg^−1^) located near Vilnius-Klaipėda motorway was also relatively lower than our results [53]. It is therefore apparent that Cd concentration in these soils is affected by car traffic.

The order of contamination factors of risk elements in the surface humus layer of both soils was as follows: Cd > Pb > Zn > Cu. In the case of Cambisol mineral layers, the order was as follows: Pb > Zn > Cu > Cd; and in the mineral layers of Luvisol was as follows: Pb > Cd > Zn > Cu. Level of soil contamination is similar to the results reported by Radziemska and Fronczyk [8]. These authors classified soils located at a distance of 0.5 m from the edge of the Warsaw Highway (E30) as moderately contaminated with Cu, Pb and Cd and moderately to heavily with Zn. The soil samples taken near Alexandria-Marsa Matruh highway (Egypt) were low contaminated with Zn, but moderately contaminated by Pb [64]. The largest number of soil samples taken from the urban complex Volos (Greece) was moderately contaminated, too [65]. According to study by Souffit et al. [66] in eastern Cameroon, up to 26.66% of the soil samples had a medium level of contamination, 56.66% had a significant level of contamination and 16.66% had a very high level of contamination.

### 4.2. Concentration of Trace Elements in Tested Plant Species

The concentration of zinc in leaves of tested species growing on Cambisol (Table 4) ranged as a rule within the range established for the background condition by Bowen [67]. The only exception is *Q. cerris* in the leaves of which a slightly higher value of 41.53 mg kg^−1^ was found. The mean concentration of Zn in foliage of all examined oaks reaches 35.98 mg kg^−1^ and is about 40% higher compared to the mean value of 25.6 mg kg^−1^ for foliage of Slovak oaks [68]. Sensitive terrestrial plants die when soil Zn concentration exceeds 100 mg kg^−1^ and photosynthesis is stopped when the concentration is more than 178 mg Zn kg^−1^ [69]. Concentrations between 30 and 100 mg Zn kg^−1^ DW are enough to support adequate plant growth, whereas toxicity symptoms are observed in concentrations above 300 mg Zn kg^−1^ DW for species that are not adapted to high-zinc exposure [70]. The sensitive plant species are reported to be retarded in growth when their tissues contain 150–200 mg Zn kg^−1^ [71]. Most often, however, the upper toxic levels range in various plants from 100 to 500 mg kg^−1^ [72].

Zinc concentration in the leaves of tested species growing on Luvisol ranged from 27.21 to 40.13 mg kg^−1^, with the exception of *A. sylvestris* species whose concentration (57.19 mg Zn kg^−1^) is slightly higher than the reference value of 50 mg Zn kg^−1^ reported by Markert [49]. Critical Zn deficiency concentrations in leaves (15–20 mg kg^−^^1^ DW) were noticed only in the leaves of *F. excelsior* growing on Cambisols.

The concentration of copper in the leaves of the tested species growing on Cambisol (Table 4) was within the range established for normal plants by Bohn et al. [57]. These values are even lower than an average concentration of 10 mg Cu kg^−1^ in plant tissue reported by Baker et al. [73]. They were also lower than the mean value of 7.27 mg Cu kg^−1^ for foliage of forest tree species in Slovakia found by Maňkovská [68]. The mean concentration of Cu in foliage of all examined oaks reaches 7.4 mg kg^−1^ and is by 26% lower compared to the value for foliage of Slovak oaks [68]. Critical deficiency levels are in the range of 1–5 mg Cu kg^−1^ DW and the threshold for toxicity is above 20–30 mg Cu kg^−1^ DW [66].

Copper concentration in the leaves of tested species growing on Luvisol ranged as a rule from 5.39 to 9.67 mg kg^−1^, with the exception of *A. sylvestris* species, with a slightly higher value of 16.50 mg Cu kg^−1^. A higher Cu concentration was again found in *Q. cerris* or *F. excelsior* leaves, the lowest in *Q. rubra* leaves. The species *A. sylvestris* thus generally appears to be the best indicator of Cu contamination of the forest ecosystems. Similar Cu concentrations in plants have also been found along the international route E-30, the Siedlce bypass [34]. Authors found the highest concentration of Cu regardless of the distance from the road in *Taraxacum* sp. (14.37 mg kg^−1^), the lowest for *Rumex acetosa* (7.55 mg kg^−1^) and *Vicia cracca* (8.17 mg kg^−1^).

The concentration of Pb in the leaves of tested species growing on Cambisol (Table 4) is lower than an average value of 10 mg kg^−1^ for plants reported by Brooks [74], but higher in comparison with a background value of 1 mg Pb kg^−1^ reported by Markert [49]. Overall, the highest Pb concentration was found in *N. aceroides* leaves, the lowest in *A. sylvestris*. Lead concentration in the leaves of tested species growing on Luvisol ranges from 1.80 to 3.55 mg kg^−1^, and is thus similar to that in the species growing on Cambisol. The highest Pb concentration was again found in *N. aceroides* leaves, the lowest in *F. excelsior* leaves. The mean concentration of Pb in leaves of all examined oaks reaches 3.04 mg kg^−1^ and is more than 69% higher compared to a mean value of 1.80 mg kg^−1^ found for foliage of Slovak oaks [68]. Lead poses a serious threat to plants due to its high toxicity. Overall, the observed amounts of Pb within the study plants were found above the background values, according to Dalenberg and van Driel [75].

The concentration of Cd in the leaves of tested species growing on Cambisol (Table 4) is being similar to the background value for plants reported by Bowen [69]. Only the values found for *A. sylvestris* (0.136–0.223 mg kg^−^^1^) and *Q. rubra* (0.139 mg kg^−1^) were significantly higher compared to the background value of 0.04 mg Cd kg^−1^ reported by Markert [49]. Cadmium concentration in the leaves of test species growing on Luvisol ranged from 0.041 to 0.297 mg kg^−1^, so it was generally higher compared to the species growing on Cambisol (on average twice). The only exception was Cd concentration in leaves of *F. excelsior*. An average concentration of Cd in the leaves of all examined oaks reaches 0.070 mg kg^−1^ and is more than 43% lower compared to a mean value of 0.12 mg kg^−1^ for foliage of Slovak oaks [68]. Slightly higher Cd values, compared to ours, were recorded on parts of the European route E30 (Siedlce) in *Arrhenatherum elatius* (0.251 mg kg^−1^) and in other grasses (0.324–0.345 mg kg^−1^) [6]. Cadmium concentrations in studied plants are, however, much lower than those found for *Plantago lanceolata* (5.7 to 13.8 mg kg^−^^1^) growing in polluted areas of Poland [76]. The phytotoxic concentrations of Cd in sensitive plant species can be within 5–10 mg kg^−^^1^ whereas the critical Cd levels range between 10–20 mg kg^−^^1^ [72].

In general, transfer of risk elements in nature is a process driven by a number of natural and anthropogenic factors. The results showed that TC values are relatively low, probably due to the short operation time of the motorway. Overall, the lowest TC values were found for Pb on both soil types. The plant roots can act as a barrier to the translocation of heavy metals within plant uptake of Pb and it is obviously related to its diminished transfer capacities from roots into leaves [77]. The same phenomenon was found also for Cu [25]. The results showed that Zn concentrations in Cambisol were significantly correlated with Zn concentrations in plants (range of r = 0.807 - 0.891). The same can be said for Cu concentrations in Luvisol (range of r = 0.442 - 0.599). Similar correlation coefficients were also found for Zn in *Vaccinium myrtillus* (r = 0.657) and *Dryopteris filix-mas* (r = 0.508), as well as for Cu in *V. myrtillus* (r = 0.546) growing on Cambisol and Podzol in NP Slovenský raj, Eastern Slovakia [78].

## 5. Conclusions

Contamination factor values showed that the surface humus layer of both soil types is moderately contaminated with Zn, low contaminated with Cu and considerably contaminated with Pb and Cd. Contamination of Luvisol surface humus layer with Pb is very high. The mineral layers of Cambisol are moderately contaminated with Zn, Cu, Pb and Cd and that of Luvisol with Pb and Cd. Contamination of Luvisol mineral layers with Zn and Cu is low. Zinc concentrations in Cambisol correlated significantly with Zn concentrations in plants, and the same can be said for Cu concentrations in Luvisol. The correlations between Pb and Cd concentrations of both soil types and the studied species were insignificant. Soil-plant transfer coefficients (TC) higher than 1 were found only for *A. sylvestris* leaves (Zn, Cu) growing on Luvisol and this species also had the highest TC for Cd on both soil units. This species therefore appears to be the best indicator of environmental pollution by car traffic.

At present, the soils and plants of the studied forest ecosystems are mostly at an early stage of contamination with risk elements. Therefore, the data obtained can be useful in the long term in assessing the development of accumulation and transfer of heavy metals from soil and air to specific plant species, as well as in screening potential plant accumulators. A sufficient understanding of the evolution of the contamination of forest ecosystems over time and space requires further research to enable effective remedial action to be taken if necessary. In the future, it will be necessary to focus on some other significant pollutants associated with the intensification of road traffic.

## Figures and Tables

**Table 1:** General geobiocoenological characteristics of forest ecosystems.

Forest Ecosystem	A *Querci-Fageta Typica*	B *Querci-Fageta Typica*
Forest vegetation grade	3rd, oak-beech	
Edaphic-hydric order	leading	wetted (semi-permeable)
Edaphic-trophic order	mesotrophic	
Soil unit	Eutric Kato-skeletic Cambisol	Eutric Kato-stagnic Luvisol
Parent rock	sandy-loam gravels of slope sediments	loess loam deposited on the fluvial sandy-gravels
Altitude (m)	203	201
Exposure	NE	–
Slope	10–15°	0°
Geographical coordinates	48°34′18.39″ N 21°14′45.20” E	48°33′22.98″ N 21°13′53.83″ E

**Table 2:** Experiment setup.

Forest Ecosystem	Soil Unit	Motorway Distance (m)	Samples of		Number of Sampled		
			Surface Humus	Mineral Layers	Plant Species	Plots (100 m^2^)	Plant Leaves
A *Querci-Fageta* Typica	Katoskeletic Cambisol	30	3	9	5	3	15 × 50
B *Querci-Fageta* Typica	Katostagnic Luvisol	30	3	9	5	3	15 × 50

**Table 3:** Selected properties of the soils. ^a^ – without skeleton; ^b^ + up to 5% skeleton.

Forest Ecosystem	Soil	Soil Layer	Humus	Skeleton	Fine Earth Fraction [mm]					pH		C/N
					<0.002	<0.01	0.01– 0.063	0.063– 0.1	0.1–2			
		[cm]	[%]							H_2_O	KCl	
A *Querci-Fageta* *Typica*	Eutric Kato-skeletic Cambisol	0–5	5.97	10–20	11.60	39.23	38.41	2.36	19.91	6.68	6.35	11.5
		10–20	3.85		11.97	38.46	38.50	3.70	19.28	5.71	5.21	12.8
		25–35	3.84	30–40	11.64	37.40	37.45	4.56	20.53	5.13	4.41	10.1
B *Querci-Fageta* *Typica*	Eutric Kato-stagnic Luvisol	0–5	5.60	− ^a^	9.32	39.91	51.14	5.47	3.42	5.90	5.71	11.1
		10–20	2.31	− ^a^	12.79	43.16	51.54	2.04	3.20	5.63	4.85	11.6
		25–35	1.18	+ ^b^	12.49	41.64	51.29	4.96	1.97	5.53	4.54	11.7

**Table 4:** The significance of the differences between the concentrations of the elements found in the leaves of the same plant species on Cambisol and Luvisol was tested by ANOVA and Fisher’s LSD test. Significantly different mean values (*p* ˂ 0.05) are indicated by different letters (^a,b^).

Forest Ecosystem	Soil Unit	Plant Species	Zn	Cu	Pb	Cd
			(mg kg^−1^ ± SD)			
A *Querci-Fageta* *Typica*	Eutric Kato-skeletic Cambisol	*Anthriscus sylvestris*	29.69 ± 8.3 ^b^	7.18 ± 1.01 ^b^	1.38 ± 0.24 ^b^	0.136 ± 0.04 ^b^
		*Fraxinus excelsior*	13.33 ± 3.7 ^b^	6.40 ± 0.90 ^b^	2.32 ± 0.41 ^a^	0.041 ± 0.01 ^a^
		*Quercus cerris*	41.53 ± 11.7 ^a^	8.86 ± 1.24 ^a^	3.50 ± 0.61 ^a^	0.041 ± 0.01 ^a^
		*Quercus rubra*	39.68 ± 11.2 ^a^	6.23 ± 0.87 ^a^	3.58 ± 0.63 ^a^	0.044 ± 0.01 ^a^
		*Negundo aceroides*	26.65 ± 7.5 ^a^	4.38 ± 0.61 ^b^	3.70 ± 0.65 ^a^	0.036 ± 0.02 ^b^
		Average	30.18 ± 8.48 ^a^	6.61 ± 1.62 ^a^	2.90 ± 1.01 ^a^	0.059 ± 0.04 ^a^
B *Querci-Fageta* *Typica*	Eutric Kato-stagnic Luvisol	*Anthriscus sylvestris*	57.19 ± 16.1 ^a^	16.50 ± 2.29 ^a^	2.41 ± 0.42 ^a^	0.297 ± 0.10 ^a^
		*Fraxinus excelsior*	29.41 ± 8.3 ^a^	9.67 ± 1.35 ^a^	1.80 ± 0.32 ^a^	0.041 ± 0.01 ^a^
		*Quercus cerris*	31.67 ± 8.9 ^a^	9.27 ± 1.30 ^a^	3.53 ± 0.63 ^a^	0.061 ± 0.02 ^a^
		*Quercus rubra*	40.13 ± 11.3 ^a^	5.39 ± 0.75 ^a^	3.06 ± 0.54 ^a^	0.083 ± 0.03 ^a^
		*Negundo aceroides*	27.21 ± 7.6 ^a^	6.19 ± 0.87 ^a^	3.55 ± 0.60 ^a^	0.106 ± 0.04 ^a^
		Average	37.12 ± 10.44^a^	9.49 ± 4.38^a^	2.87 ± 0.76^a^	0.117 ± 0.10^a^
Reference values according to Markert [49]			50	10	1	0.05

**Table 5:** Transfer coefficients of trace elements found for foliage of studied plant species.

Forest Ecosystem	Soil Unit	Risk Element	*Anthriscus* *sylvestris*	*Fraxinus* *excelsior*	*Quercus* *cerris*	*Quercus* *rubra*	*Negundo* *aceroides*
A *Querci-Fageta* *Typica*	Eutric Kato-skeletic Cambisol	Zn	0.33 ± 0.06	0.15 ± 0.02	0.46 ± 0.08	0.44 ± 0.08	0.30 ± 0.05
		Cu	0.35 ± 0.02	0.32 ± 0.02	0.44 ± 0.08	0.31 ± 0.02	0.22 ± 0.01
		Pb	0.05 ± 0.01	0.08 ± 0.02	0.08 ± 0.01	0.12 ± 0.01	0.12 ± 0.01
		Cd	0.35 ± 0.01	0.11 ± 0.01	0.11 ± 0.01	0.12 ± 0.01	0.09 ± 0.03
B *Querci-Fageta* *Typica*	Eutric Kato-stagnic Luvisol	Zn	1.07 ± 0.13	0.55 ± 0.07	0.59 ± 0.07	0.75 ± 0.09	0.51 ± 0.06
		Cu	1.60 ± 0.07	0.94 ± 0.02	0.90 ± 0.04	0.52 ± 0.02	0.60 ± 0.02
		Pb	0.08 ± 0.01	0.06 ± 0.01	0.11 ± 0.01	0.10 ± 0.01	0.11 ± 0.01
		Cd	0.69 ± 0.01	0.10 ± 0.01	0.14 ± 0.01	0.19 ± 0.01	0.24 ± 0.01

**Table 6:** Pearson’s correlation coefficients (r) between trace elements in soils and plants (*n* = 12). * *p* ˂ 0.05; ** *p* ˂ 0.01.

Soil Unit	Cambisol				Luvisol			
Plant/Element	Zn	Cu	Pb	Cd	Zn	Cu	Pb	Cd
*Anthriscus sylvestris*	0.807 **	0.335	0.107	0.135	0.208	0.544 **	0.131	0.208
*Fraxinus excelsior*	0.891 **	0.299	0.137	0.078	0.199	0.599 **	0.096	0.161
*Quercus cerris*	0.854 **	0.369	0.173	0.127	0.195	0.457 *	0.089	0.112
*Quercus rubra*	0.831 **	0.352	0.120	0.193	0.201	0.442 *	0.136	0.183
*Negundo aceroides*	0.835 **	0.336	0.146	0.166	0.198	0.460 *	0.170	0.156

## Data Availability

Data set available on request to corresponding authors.
